# Accuracy of Interpolated Versus In-Vineyard Sensor Climate Data for Heat Accumulation Modelling of Phenology

**DOI:** 10.3389/fpls.2021.635299

**Published:** 2021-07-13

**Authors:** Paula Pipan, Andrew Hall, Suzy Y. Rogiers, Bruno P. Holzapfel

**Affiliations:** ^1^School of Agriculture and Wine Science, Charles Sturt University, Wagga Wagga, NSW, Australia; ^2^National Wine and Grape Industry Centre, Charles Sturt University, Wagga Wagga, NSW, Australia; ^3^Institute for Land, Water and Society, Charles Sturt University, Albury, NSW, Australia

**Keywords:** viticulture, climate change, phenology, climatic indices, climate data

## Abstract

**Background and Aims:**

In response to global heating, accurate climate data are required to calculate climatic indices for long-term decisions about vineyard management, vineyard site selection, varieties planted and to predict phenological development. The availability of spatially interpolated climate data has the potential to make viticultural climate analyses possible at specific sites without the expense and uncertainty of collecting climate data within vineyards. The aim of this study was to compare the accuracy and precision of climatic indices calculated using an on-site climate sensor and an interpolated climate dataset to assess whether the effect of spatial variability in climate at this fine spatial scale significantly affects phonological modelling outcomes.

**Methods and Results:**

Four sites comprising two topographically homogenous vineyards and two topographically diverse vineyards in three wine regions in Victoria (Australia) were studied across four growing seasons. A freely available database of interpolated Australian climate data based on government climate station records (Scientific Information for Land Owners, SILO) provided temperature data for grid cells containing the sites (resolution 0.05° latitude by 0.05° longitude, approximately 5 km × 5 km). In-vineyard data loggers collected temperature data for the same time period. The results indicated that the only significant difference between the two climate data sources was the minimum temperatures in the topographically varied vineyards where night-time thermal layering is likely to occur.

**Conclusion:**

The interpolated climate data closely matched the in-vineyard recorded maximum temperatures in all cases and minimum temperatures for the topographically homogeneous vineyards. However, minimum temperatures were not as accurately predicted by the interpolated data for the topographically complex sites. Therefore, this specific interpolated dataset was a reasonable substitute for in-vineyard collected data only for vineyard sites that are unlikely to experience night-time thermal layering.

**Significance of the Study:**

Access to accurate climate data from a free interpolation service, such as SILO provides a valuable tool tomanage blocks or sections within vineyards more precisely for vineyards that do not have a weather station on site. Care, nevertheless, is required to account for minimum temperature discrepancies in topographically varied vineyards, due to the potential for cool air pooling at night, that may not be reflected in interpolated climate data.

## Introduction

The concept of “terroir” has long been applied to wine regions. It is a French word, which can be defined as “an elusive combination of the effects of sun, soil, weather and history” Deloire (2008). In Australia, there is a rapidly growing movement and consumer demand for regionally authentic and recognisable wines. Quantifying the components that contribute to the distinct, recognizable flavour profile of a wine from a specific region or indeed from a specific vineyard is important. This quantification will aid in understanding how, if possible, to mitigate the effects of global heating in order to sustain that distinct, recognisable and marketable flavour profile. Of all the aspects of terroir contributing to wine flavour, climate has been found to have the greatest effect (Webb et al., 2008; Bonada et al., 2015; Pons et al., 2017; Geffroy et al., 2019), because the stages of grapevine growth (phenology) are driven by climate, or more specifically by temperature (Gladstones, 1992, 2011; Jones and Davis, 2000).

Climate is driven by the amount of solar radiation (insolation) received by a surface (Oke, 2002), hence the latitude, altitude, slope, and aspect of a vineyard site will influence the insolation and therefore the climate it experiences (Jacquet and Morlat, 1997; Gladstones, 2011; Neethling et al., 2019). The amount of insolation that reaches a surface depends on the angle (slope) of that surface (Jones, 2007). Slope and aspect are interconnected. The aspect of a slope will determine how much insolation it receives so the aspect that most directly faces the sun, receives the most insolation (Oke, 2002; Jones, 2007). Water availability, insolation, and temperature are the main drivers of photosynthesis in the grapevine which controls the production of carbohydrates and the phenological stages after budbreak (Medrano et al., 2003; Holzapfel and Smith, 2012). They also influence soil temperature, which mediates post-harvest carbohydrate accumulation (Holzapfel and Smith, 2012; Hall et al., 2016) and enhanced vegetative and reproductive growth (Field et al., 2009; Rogiers et al., 2011, 2014; Clarke et al., 2015). Heat accumulation over time determines phenological stages. In vineyards worldwide, the advancement of phenological stages has been observed due to climate change (Caffarra and Eccel, 2011; Bonnefoy et al., 2013; Malheiro et al., 2013; Cola et al., 2017; Jarvis et al., 2017; Alikadic et al., 2019).

The categorisation of the climate of a vineyard site, referred to as a mesoclimate (Coombe and Dry, 1988) or a wine region, referred to as a macroclimate (Coombe and Dry, 1988) uses a number of climatic indices. Climatic indices combine daily temperature data to produce a single index figure, which can then be categorised. These categories have been developed for grape growers to determine the suitability of a site for the growth habits and phenological development of a particular grape variety. The mapping at a macroclimate scale of climatic indices of wine growing regions has been undertaken in numerous studies around the world (Tonietto and Carbonneau, 2004; Jones et al., 2009; Hall and Jones, 2010; Irimi et al., 2014; Remenyi et al., 2019). The categorisation of viticultural regions enables the identification of climate analogues, i.e., identification of locations whose historical climate is similar to the anticipated future climate at a reference location (Grenier et al., 2013). Climate analogues have been identified by Australian grape growers as being useful when making long-term vineyard management decisions (6 to 10 years) (Dunn et al., 2015).

Temporal variation in climate or climate variability is often used to denote deviations of climatic statistics over a given period of time (e.g., a month, season or year) when compared to long-term statistics for the same calendar period. The World Meteorological Organisation (2019) defines it as variations in the mean state and other statistics of the climate on all temporal and spatial scales, beyond individual weather events. Care is required when comparing seasonal climate data to climatic index categories, as season to season climate variability can be quite extensive (Hall and Blackman, 2019).

However, spatial variation within a vineyard has been identified as being directly related to the flavour profile of wines (Marais et al., 2001; Bramley et al., 2011; Scarlett et al., 2014). Vineyards on steep sites experience thermal layering at night, as by day the earth’s surface is heated by the sun so there is thermal mixing by convection as an upward transfer of heat from the warmed surface to the cooler atmosphere occurs. By night, when the earth’s surface cools rapidly, heat is transferred downward which suppresses mixing and the formation of cold layers near the surface is observed (Oke, 2002). Therefore, climate data at a macroclimate scale are unlikely to provide accurate climatic indices at the fine spatial scale of a specific vineyard site, particularly if it is topographically varied. Hence, for long-term decisions about vineyard management, varieties to be planted, change of training system, row orientation, vineyard sites and to predict phenological development, accurate climate data at a mesoclimate scale are required to calculate climatic indices. Interpolated climate databases are available worldwide that provided climate data at the mesoclimate scale (Hijmans et al., 2005; Spittlehouse, 2006; Mbogga et al., 2010; Moreno and Hasenauer, 2016; Wang et al., 2016; Fick and Hijmans, 2017). This study investigated two sources of climate data in Australia and compared their capacity to categorise climatic indices in vineyards with both homogenous topography (open, flat plain) and diverse topography (at elevation with multiple angles of slopes and aspects). Vineyards, or other agricultural enterprises in other parts of the world could verify results in their location with local interpolated climate databases.

## Materials and Methods

### Locations and Vineyards

**FIGURE 1:**
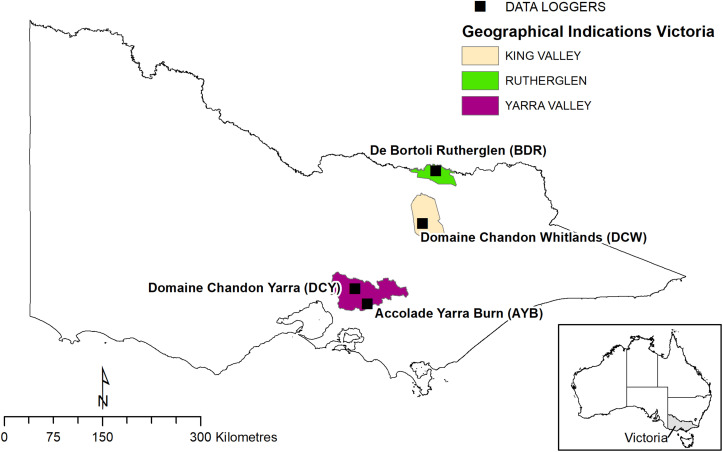
Map with location of vineyards marked.

**FIGURE 2:**
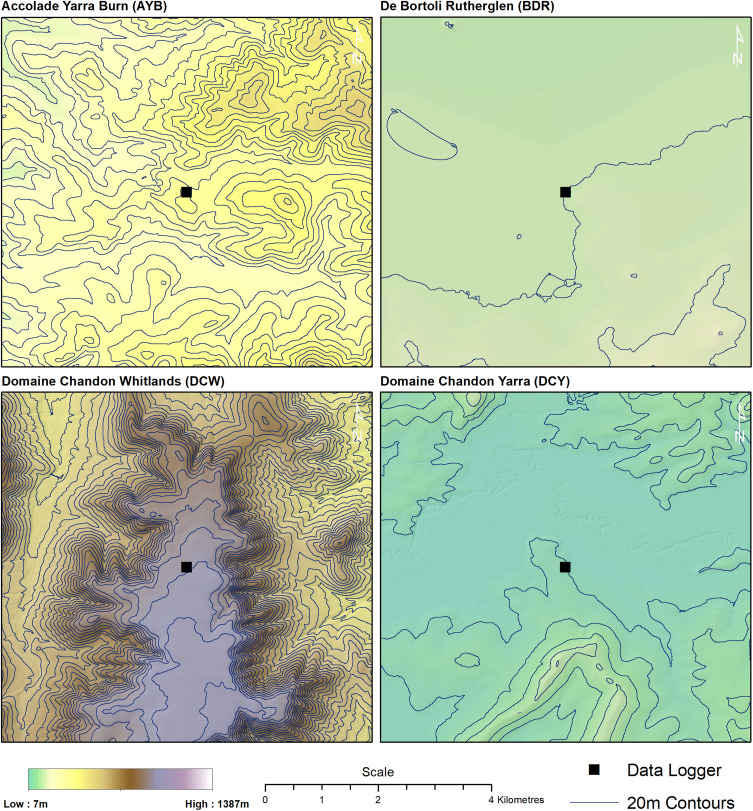
Digital elevation model of vineyard sites.

Four vineyard sites were selected for this study in three Victorian (Australia) wine regions, varying in topographic complexity (Figures 1, 2).

Two vineyards were classified as topographically diverse (TD):

1. Accolade Yarra Burn (AYB) Beenak Road Vineyard, Hoddles Creek (Yarra Valley GI), which is a steep hilly site with a top elevation of 446 m. Coordinates -37.887S, 145.601E
2. Domaine Chandon (DCW), Mansfield-Whitfield Road, Whitlands Vineyard (King Valley GI), which is an undulating site with a top elevation of 790 m. Coordinates -36.781S, 146.360E

Another two vineyards were classified as topographically homogenous (TH):

3. Domaine Chandon (DCY), Maroondah Hwy Vineyard, Coldstream (Yarra Valley GI) which is a relatively even, flat valley site at an elevation of 150 m. Coordinates -37.677S, 145.431E
4. De Bortoli (DBR) (formerly Rutherglen Estates), Great Northern Road vineyard, Rutherglen (Rutherglen GI) which is a relatively even, flat wide valley site at an elevation of 150 m. Coordinates -36.055S, 146.540E

The cultivars grown in the case study vineyards are those most suited to the climate of that vineyard. Hence, the data logger placement was in Chardonnay blocks for three of the vineyards: AYB, DCW, and DCY. The DBR vineyard is a much warmer site, where no Chardonnay is grown, so the data logger was in a Shiraz block. Since the study is mainly concerned with within vineyard differences caused by varying data sources, it is unlikely the different variety at DBR will significantly impact the overall results or conclusions around spatial variability in climate that can be drawn from the study.

All sites are irrigated, so it was assumed that vine water status was optimally maintained, minimising soil effects on vine water status and vine growth.

### Data Loggers

A Tinytag TGP-4500 data logger (TT) from Gemini Data Loggers Ltd (Chichester, West Sussex, United Kingdom) (calibrated by manufacturer) housed in a Stevenson screen was attached to the tops of trellising posts in the middle of each of the four vineyards recording temperature, humidity and dewpoint every 30 min for four consecutive growing seasons of 2015-16, 2016-17, 2017-18, and 2018-19.

### Interpolated Climate Data Source

Scientific Information for Landowners (SILO) climatic data corresponding to the above four vineyard sites for the four growing seasons of the study was downloaded. Scientific Information for Landowners uses temperature data from 4600 Australian Bureau of Meteorology (BOM) weather stations and applies smoothing splines to generate interpolated surfaces on a regular 0.05^*o*^ grid (approximately 5 km × 5 km) of Australia (Jeffrey et al., 2001) with latitude, longitude and elevation as independent variables. SILO data is freely available and easily accessible to grape growers from https://www.longpaddock.qld.gov.au/silo/. It is acknowledged that the 5 km × 5 km pixel may present issues in adequately accounting for the spatial variation within the pixel, in particular at the higher altitude and topographically varied vineyard sites at AYB and DCW. Scientific Information for Landowners data contains measurements for many climatic indicators, but for this project, maximum and minimum air temperature SILO data were used.

### Climate Summaries

The BOM climate summaries archive for the state of Victoria gave the following descriptions of the four growing seasons monitored in this study (Table 1):

**TABLE 1:** Victorian climate descriptions for the four seasons of the study from the Bureau of Meteorology.

	Spring (Sept–Nov)	Summer (Dec–Feb)	Autumn (Mar–May)
2015–16	Rainfall 47% below average of 181 mm	Rainfall near average of 120 mm	Rainfall near average of 156.8 mm
	Mean temp 2.05 °C above long-term average	Mean temp +1.73 °C above long-term average	Mean temp 1.88 °C above long-term average, highest on record
2016–17	Rainfall 42% above average of 181 mm	Rainfall 7% below average of 120 mm	Rainfall near average of 156.8 mm
	Mean temp 0.10 °C below long-term average	Mean temp 0.87 °C above long-term average	Mean temp 1.08 °C above long-term average.4th warmest autumn on record
2017–18	Rainfall slightly below average of 181 mm	Rainfall 6 % above average of 120 mm	Rainfall 39.2% below average of 156.8 mm
	Mean temp 1.64 °C above long-term average	Mean temp above average in top 10% of all summers on record.	Mean temp 1.17 °C above long-term average
2018–19	Rainfall 42.7% below average of 181 mm	Rainfall 12% below average of 120 mm	Rainfall 21% below average of 156.8 mm
	Mean temp 0.86 °C above long-term average	Mean temp 2.54 °C above average, highest on record	Mean temp 1.04 °C above long-term average

### Climate Data Analysis

The research produced 16 sets of minimum and maximum temperature data (four vineyards, four growing seasons) from two sources: SILO and in-vineyard Tinytag (TT) data logger. These data were analysed, and climatic indices were calculated using Excel 2016 and RStudio1.3 software as described below.

1. The growing season average minimum (GSminTave) and maximum (GSmaxTave) temperatures for the seven months from October to April from each study year at each site were calculated from the in-vineyard TTdata logger and from SILO data.
2. The growing season average minimum and maximum temperatures recorded by the in-vineyard TT data logger and interpolated by SILO from each study year at each site was analysed with Student’s *t*-test to determine if there was a statistically significant difference (*P* ≤ 0.05) between them.
3. Daily minimum and maximum temperatures for each site for each season from both in-vineyard TT data and SILO data were then used to calculate the climatic indices as listed below.
  a. Average growing season temperatures (GSTavg): the mean air temperature of all days between October 1 and April 30 (Jones, 2006), which were categorised according to Jones (2006).
  b. Growing degree days (GDD): the summation of daily average air temperature above 10°C during the 7 month growing season from October to April (Amerine and Winkler, 1944) were calculated using the following formula:
    ΣGDD10=max[(T+maxT)min/2-10,0]
    These results were categorised into the Winkler Index for the classification of wine growing regions.
  c. Heliothermal index of Huglin (HI). The summation of daily average air temperature above 10°C during the six months of the growing season from October 1 to March 31 in the southern hemisphere, incorporating a length of day coefficient with the addition of a latitude correction factor, K (Huglin, 1978).
    $$\text{HI}=\sum_{d=1}^{n}\text{max}\,\left(\left(T_{m\,e\,a\,n}-10+T_{m\,a\,x}-10\right)/2,0\right)\,K$$
    The in-vineyard derived and SILO derived HI were compared and classified according to Huglin (1978).
  d. Mean January Temperature (MJT) is the mean temperature of the warmest month (January in the southern hemisphere), classified according to Smart and Dry (1980). Mean January Temperature is well correlated with GDD. The in-vineyard TT derived and SILO derived MJT were compared and classified according to Smart and Dry (1980).

### Phenology

Two grapevine phenological stages were recorded for all four vineyards in each of the four growingseasons of the study.

1. Budbreak: was defined as the date when 50% of vines reached stage four of the modified EL system (Coombe, 1995), when green leaf tips are visible on buds. Daily vineyard observations by vineyard staff determined this stage. It is acknowledged that these observations by different staff at the four sites may vary and as such are a potential source of error. The budbreak dates were compared to regional average phenological dates determined by Hall et al. (2016). The regional predicted dates are based on three scenarios: a 1975–2004 base period using climate records, and two projected climate scenarios described in terms of the mean temperature anomalies (MTA) from the base period, i.e., +1.26 and +2.61°C. Mean temperature anomalies are the spatially average temperature increase across Australia for specific future scenarios from a selected global climate model (Hall et al., 2016).
2. Maturity: this is usually defined as modified EL stage 38 (Coombe, 1995). For this study, harvest dates were used to determine maturity. It is acknowledged that the use of harvest dates to determine maturity is a potential source of error. The harvest dates were compared to regional average phenological dates determined by Hall et al. (2016). The regional predicted dates are based on the three scenarios as described for budbreak (above).

The calculated climatic indices provided information on heat accumulation which drives the phenological stages of budburst and maturity. Comparison of the climatic indices determined whether those calculated from the SILO interpolated data matched those from the in-vineyard TT data logger. The phenological stages of budburst and maturity were compared to regional average phenological dates to determine whether they were within the base period ranges or within either of the two projected climate change scenarios. In the currently warmest wine grape-producing regions, the warming trend will likely lead to the ripening period taking place earlier, in a warmer part of summer, which, in addition to the general pattern of warming, can greatly accelerate ripening leading to a potential loss of fruit quality and wine value. Jones (2015) refers to the balance of the four ripeness clocks of sugar accumulation, acid respiration, phenolic ripeness, and fruit character being disrupted by this warming pattern. This has been seen in other studies (Boss et al., 2014; Gaiotti et al., 2018; Geffroy et al., 2019).

## Results

### Seasonal Temperatures

**FIGURE 3:**
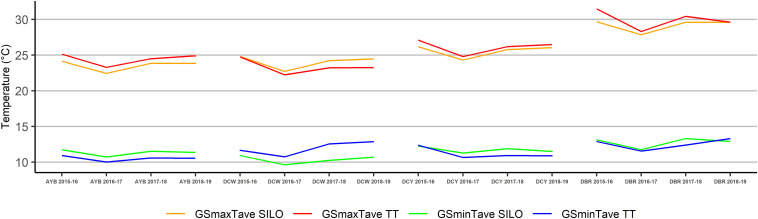
Average growing season minimum temperature (GSminTave) and average growing season maximum temperature (GSmaxTave) of four sites across four seasons as calculated using Tinytag (TT) or SILO data.

During the four growing seasons of the study in the two topographically diverse (TD) vineyards, the average growing season minimum temperature (GSminTave) at Accolade Yarra Burn (AYB) were 0.7 to 0.9°C lower for the in-vineyard TT data logger results than for SILO results but were 0.7 to 2.3°C higher at Domaine Chandon Whitlands (DCW) (Figure 3). The GSminTave in the both of the topographically homogenous vineyards (TH) at Domaine Chandon Yarra (DCY) and De Bortoli Rutherglen (DBR) showed no consistent trend between the in-vineyard TT data logger results and the SILO results (Figure 3).

For the four seasons of the study, the maximum (GSmaxTave) temperatures for the in-vineyard TT data logger results were 0 to 1.8°C higher than the SILO results for both the TH vineyards at DCY and DBR and the TD vineyard at AYB. However, they were 0 to 1.0°C lower at the TD vineyard at DCW (Figure 3).

GSminTave in the TD vineyards of AYB and DCW showed significant differences (*P* ≤ 0.05) using Student’s *t*-test between the in-vineyard measured and SILO interpolated data (Table 2). For the TH vineyards, there were no statistically significant differences in minimum temperatures except at DCY in the hot 2017–18 growing season.

**TABLE 2:** Comparing average growing season minimum and maximum temperature data sets (TT, SILO) for four sites and four growing seasons.

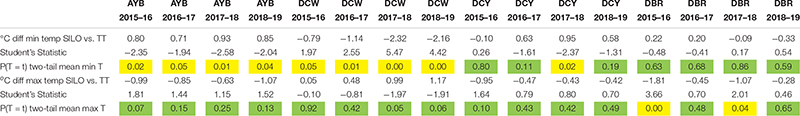

*Yellow indicates statistically significant difference; green indicates no statistically significant difference.*

The Student’s *t*-test results for the average growing season maximum temperatures only showed significant differences (*P* ≤ 0.05) between the in-vineyard data loggers and SILO data in the TH DBR vineyard in the two warmer seasons of 2015–16 and 2017–18, when in-vineyard temperatures showed consistently higher values (Table 2).

### Climatic Indices

**FIGURE 4:**
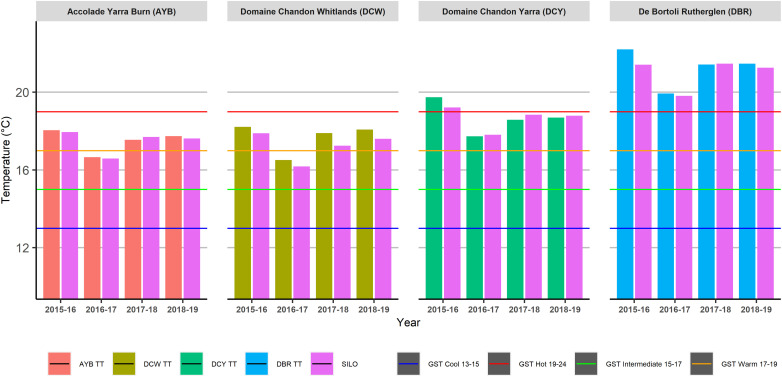
Average Growing Season Temperatures (GSTavg) of four sites across four seasons as calculated using Tinytag (TT) or Scientific Information for Landowners (SILO) data.

All sites for the four seasons of the study were categorised in the same viticultural classification for average growing season temperature (GSTavg) (Figure 4) for both the in-vineyard TT data and the SILO data. Despite the topographically diverse vineyards having significant differences in average minimum temperatures, this did not influence the GSTavg classifications.

However, the classifications showed temporal variations. In the cool growing season (2016–17), in both of the TD vineyards at AYB and DCW the GSTavg classification was “Intermediate” but was “Warm” in the other three growing seasons. For the TH vineyard at DCY, GSTavg classification in the hot growing season (2015–16) was “Hot” but was “Warm” in the other three growing seasons. De Bortoli Rutherglen (DBR) remained in the “Hot” classification across all four growing seasons.

**FIGURE 5:**
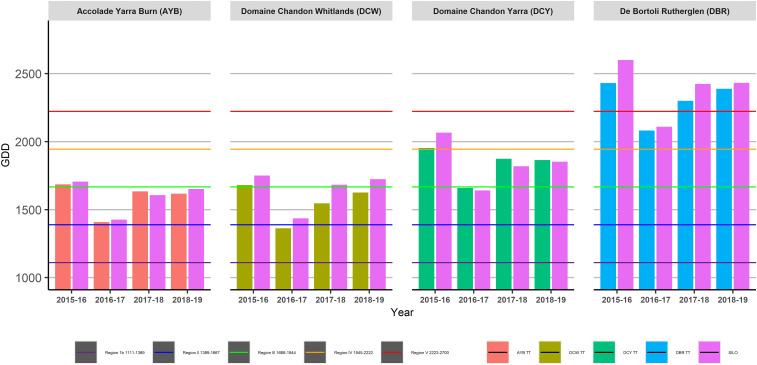
Growing Degree Days (GDD) for four sites across four growing seasons as calculated using Tinytag (TT) or Scientific Information for Landowners (SILO) data.

In the TD vineyard at AYB, the GDD classification based on Winkler (Figure 5) remained the same within growing seasons for both in-vineyard TT and SILO data. However, it was a Region III classification in the hot growing season (2015–16) and Region II in the other three seasons of the study.

The TD DCW vineyard had a higher GDD classification (Region II) in the cool season (2016–17) for in-vineyard TT data compared to SILO data (Region Ib) and also for the 2017–18 and 2018-19 seasons where in-vineyard TT data gave a classification of Region III compared to Region II with SILO data. The hot growing season (2015–16) showed classification of Region III for both data sets.

The TH DCY vineyard showed consistent GDD classifications between in-vineyard TT data logger and SILO data within the same season, but changed from Region IV in the hot 2015–16 season, to Region II in the cool season (2016–17) and to Region III in the intermediate seasons (2017–18 and 2018–19).

The TH DBR vineyard classifications were consistent between in-vineyard TT and SILO data within the same season; however, the classification changed from Region IV in the cooler season (2016–17) to Region V in the other three seasons.

**FIGURE 6:**
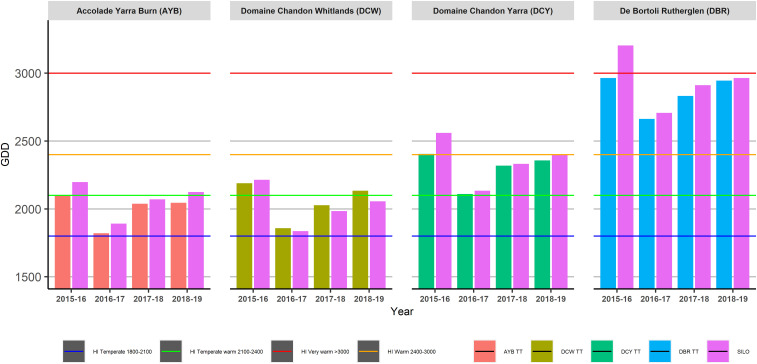
Heliothermal index of Huglin (HI) across four sites and four seasons as calculated using Tinytag (TT) or Scientific Information for Landowners (SILO) data.

The TH vineyards at DCY and DBR showed consistent classifications within the same season for Heliothermal index of Huglin (HI) for both data collection methods, except for the hotter 2015–16 season at DBR where TT data gave a “Very Warm” classification and SILO gave a “Warm” classification (Figure 6). Huglin classification remained consistent in both TH vineyards for all seasons, except the hotter growing season (2015–16).

The HI classifications in the TD vineyards at AYB and DCY remained the same for both sets of data in the cooler season (2016–17) and the intermediate growing season (2017–18). However, in the other seasons (2015–16 and 2018–19), the SILO data gave a cooler HI classification than the TT data at AYB, and a warmer HI classification in 2018–19 at DCY.

**FIGURE 7:**
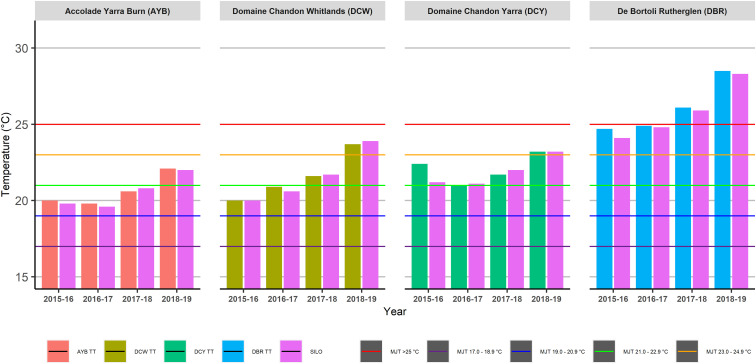
Mean January Temperature (MJT) across four sites and four seasons as calculated using Tinytag (TT) or Scientific Information for Landowners (SILO) data.

Mean January Temperature (MJT) results were consistent between in-vineyard TT data and SILO data within the same season for all sites across all four growing seasons, although all sites recorded their highest MJT in the 2018–19 season (Figure 7).

### Phenology

Topographically diverse (TD) AYB vineyard had consistent, early budbreak dates in the second week of September, across the four growing seasons (Table 3), with a range of five days between the earliest and latest budbreak dates. The highest elevation vineyard at DCW (790 m) recorded late budbreak dates in early to mid-October each year, with a fair degree of variation between the earliest and latest budbreak dates (15-day range over the four seasons). The TH vineyards at DCY and DBR showed a large variation in budbreak dates from early September to early October between growing seasons (13 days and 24 days, respectively), with the earliest budbreak at both vineyards occurring in the cooler (as calculated by heat accumulation) 2016–17 season.

**TABLE 3:** Budbreak and maturity (harvest) dates in number of days from July 1 and number of days between both stages. Ch = Chardonnay, Sh = Shiraz.

Vineyard	Budbreak (No. of days from July 1)	Harvest date (No. of days from July 1)	No. of days between budbreak and harvest
AYB 2015–16 (Ch)	71	240	169
AYB 2016–17 (Ch)	71	251	180
AYB 2017–18 (Ch)	76	257	181
AYB 2018–19 (Ch)	76	240	164
DCW 2015–16 (Ch)	101	245	143
DCW 2016–17 (Ch)	113	258	144
DCW 2017–18 (Ch)	106	249	142
DCW 2018–19 (Ch)	98	245	147
DCY 2015–16 (Ch)	79	227	148
DCY 2016–17 (Ch)	68	242	174
DCY 2017–18 (Ch)	81	228	147
DCY 2018–19 (Ch)	72	237	165
DBR 2015–16 (Sh)	83	230	147
DBR 2016–17 (Sh)	74	247	173
DBR 2017–18 (Sh)	98	246	148
DBR 2018–19 (Sh)	91	241	150

Accolade Yarra Burn (AYB) had a range of 17 days between the earliest and latest harvest dates over the four seasons; DCW had a range of 14 days; DCY 15 days, and DBR 17 days (Table 3).

The number of days between budbreak and maturity/harvest date (Table 3) for the two TH vineyards at DCY and DBR showed a marked increase in the cooler 2016–17 season, but no clear pattern was observed in the TD vineyards at AYB and DCW. The highest elevation and cooler vineyard at DCW had a consistent length of vintage, with only a five-day difference in number of days between budbreak and harvest across the four seasons. Topographically diverse vineyard AYB had a 16-day difference between its longest and shortage vintage, and the TH vineyards at DCY and DBR had large differences in vintage length at 27 and 26 days, respectively.

Budbreak dates were compared to the regional predicted dates after Hall et al. (2016) (Table 4). Both the TH and TD vineyards in the Yarra Valley (DCY and AYB, respectively) had budbreak dates between the minimum and maximum values of the MTA 1.26 scenario. The TD and highest elevation vineyard at DCW was within the minimum and maximum modelled dates without an MTA scenario applied. The TH DBR vineyard had the greatest range of budburst, coinciding with the MTA 1.26 scenario in the 2018–19 season, and the MTA 2.61 scenario in the 2016–17 season.

**TABLE 4:** Modelled budbreak dates (number of days from July 1) using a 1975–2004 base period and projected mean temperature anomalies (MTA) of 1.26 and 2.61°C (after Hall et al., 2016).

Region	Min	Q1	Median	Q3	Max
Yarra	75	82	86	89	93
Yarra MTA1.26	66	73	76	79	82
King Valley	93	94	98	105	113
Rutherglen	90	93	96	98	114
Rutherglen MTA1.26	80	83	85	88	100
Rutherglen MTA2.61	71	73	75	77	88

Harvest dates were compared to the regional predicted dates after Hall et al. (2016) (Table 5). The TD vineyard in the Yarra Valley, AYB had harvest dates coincident with the MTA 1.26 scenario. Harvest dates in the TH Yarra vineyard, DCY coincided with both the MTA 1.26 and the MTA 2.61 scenarios. The TD and highest elevation vineyard at DCW was within the maturity dates determined for the 1974–2005 base period. The TH DBR vineyard had harvest dates coincident with the MTA 1.26 scenario.

**TABLE 5:** Modelled maturity dates (number of days from July 1) for base period 1975–2004 and projected mean temperature anomalies (MTA) of 1.26 and 2.61**°**C (after Hall et al., 2016).

Region	Min	Q1	Median	Q3	Max
Yarra	259	277	284	297	329
Yarra MTA1.26	237	249	252	258	265
Yarra MTA2.61	220	230	232	236	240
King Valley	242	246	254	272	305
Rutherglen	233	237	242	247	281
Rutherglen 1.26	219	223	227	230	250
Rutherglen 2.61	205	209	212	216	231

## Discussion

Scientific Information for Landowners (SILO) interpolation generates climate maps for Australia by applying smoothing splines to data at weather station locations on a 0.05^*o*^ grid (Jeffrey et al., 2001). Interpolations at this scale are unable to represent fine spatial variability in climate for topographically complex sites at the within vineyard management unit scale.

### Temperatures

The main significant differences that were found when comparing the data from the two sources were in the minimum temperatures in the topographically diverse vineyards at AYB and DCW. This would be consistent with the thermal layering that would occur at night in these vineyards (Oke, 2002) which would make interpolations of minimum temperatures more difficult on the hilly sites where there would be numerous layers at different temperatures. Due to daytime thermal mixing, maximum daily temperatures would be vertically homogenous and therefore more accurately interpolated by SILO in both topographically homogenous and topographically diverse areas. This was found to be the case (Table 2) with maximum daily temperatures not differing significantly from each other. Of note was the direction of the difference in minimum temperatures between TT and SILO data. Scientific Information for Landowners overestimated the minimum temperatures at AYB and underestimated the minimum temperatures at DCW. Modelling at this scale uses a broad environmental lapse rate (a rate of temperature change with respect to elevation) and cool air pooling cannot be represented at the within-vineyard scale. Accolade Yarra Burn was topographically the most complex site, as can be seen in Figure 2 compared to the high plateau of DCW. Cool air pooling and multiple thermal layers were more likely at AYB, resulting in observed minimum temperatures being lower than those interpolated by SILO. At the undulating high plateau at DBW, the potential exists for warmer than interpolated minimum temperatures due to flatter terrain retaining greater heat than sloping sites (Oke, 2002; Jones, 2007).

No correlation was found between the coolness of the day and the magnitude of the difference between the minimum temperatures recorded by the data logger and that interpolated by SILO (data not shown). The lack of correlation is probably due to weather factors that influence cool air pooling, such as clear atmospheric conditions when the ground becomes relatively cooler than the air temperature on that day (Oke, 2002, p. 180). This can happen at any level of minimum temperature, not just on cool days. Another weather factor that can affect cool air pooling is wind speed, with more geostrophic wind resulting in a mixed boundary layer, preventing cool air pooling at the surface. Day to day weather, therefore, is more likely to be a factor that determines the level of cool air pooling than simply an assessment of minimum temperatures.

### Climatic Indices

Daily temperature data from both data sources were used to calculate heat accumulation climatic indices commonly employed in viticulture to make long-term vineyard management decisions. The two topographically diverse vineyards at AYB in the Yarra Valley and DCW in the King Valley were also the cooler sites in this study, based on their GSTavg (Figure 4), GDD (Figure 5), HI (Figure 6), and MJT (Figure 7). It had been expected that climatic indices calculated from SILO climate data would be more likely to closely match climatic indices calculated from data collected in-vineyard for TH vineyards (DCY and DBR) than TD vineyards at higher elevations (AYB and DCW). Furthermore, it was expected that the calculation of the climatic indices in the TD vineyards may have resulted in different classifications from the two sources of data (Figures 4–7) within the same season. In fact, the classifications did differ within the same season for GDD in the highest elevation and TD vineyard at DCW for three of the seasons studied and with the HI for both of the TD vineyards for two of the seasons. As expected, the climatic indices calculated from both sources at the topographically homogenous vineyards at DCY and DBR had consistent classifications of climatic indices within the same season, with the exception of HI at DBR in the highest heat accumulation season in 2015–16.

### Temporal Variation

Temporal variation between growing seasons was greater than any spatial scale differences observed in the vineyards. As evidenced in other studies (Holzapfel and Smith, 2012; Hall and Blackman, 2019; Priori et al., 2019), the dominant effect of climate variability due to seasonal changes in weather patterns is not unusual. The greatest heat accumulation occurred in the 2015–16 season when all four sites recorded their highest GSTavg, GDD, and HI. The lowest heat accumulation occurred in the 2016–17 season while the other two seasons tracked slightly cooler than 2015–16. The MJT was warmest at all four sites in 2019. This is consistent with the BOM climate summaries (Table 1) where the warmest summer on record was in 2019. These temporal variations were also noted when the results were compared to average indices calculated regionally, over 30 years (Hall and Jones, 2010; Jarvis et al., 2017) (data not shown). Climatic indices GSTavg, GDD, and HI at the TH sites at DCY and DBR were higher than the regionally calculated indices for all seasons except the cooler 2016–17. In contrast the TD vineyards at ABY and DCW were within the Hall and Jones (2010) ranges compared to the single averages given by Jarvis et al. (2017). This would indicate that as noted by Jones (2006), warmer vineyard regions can expect the effects of global warming to be more significant than in cooler regions.

### Phenology and Climate Change

#### Budbreak

It has been found that the actual bud temperature, rather than air temperature drives the timing of budbreak (Keller and Tarara, 2010). This would be related to the amount of insolation received by the plant, which is dependent on the latitude, altitude, slope and aspect of the vineyard site (Jacquet and Morlat, 1997; Gladstones, 2011; Neethling et al., 2019). There is the added complexity of differing budbreak heat sums for clones of the same variety (Ladányi et al., 2010), for different rootstocks (Jogaiah et al., 2013) and for viticultural practices such as late pruning (Silvestroni et al., 2018) which all influence the required heat accumulation for budbreak. It needs to be acknowledged that in an ideal situation all vineyard management practises including pruning dates for the four study vineyards would have been the same but considering these were commercial vineyards this was not possible. Aside from temperature, these inconsistent practises will likely have impacted to some extent on the results. For example, increasingly later pruning into early spring had been adopted at the TH vineyard at DBR to delay budbreak. The 2015–16 season was pruned in mid-August, the following three seasons were each pruned a week later than the year before (M. Partridge, vineyard manager, personal communication, February 18, 2020). However, delaying pruning had no consistent effects on date of budburst. This highlights the dominant effect of climate variability from season to season (Hall and Blackman, 2019) on heat accumulation, therefore affecting phenological development.

#### Maturity

In this study, the actual harvest dates were used in the comparison with predicted maturity dates of Hall et al. (2016). This is a potential source of error, as harvest date is determined by wine style, and not determined by EL stage 38 (Coombe, 1995). The three Chardonnay vineyards at AYB, DCW and DCY were harvested quite early for sparkling wine, which requires lower sugar ripeness (around 10.5 Baumé) and higher acid levels (Rankine, 2007) than traditional table wine. A regionally acquired prediction model based on table wine maturity may not be able to fully account for the early picking for sparkling wine. Similarly, at DBR, the winemaker explained that although the sugar ripeness in the Shiraz may well have been reached earlier, the flavour and tannin ripeness may not have been achieved. He stated that they usually harvested at 14.2 to 14.5 Baumé in order to avoid “green” flavour and tannins (M. Scalzo, personal communication February 18, 2020). This is corroborated by Hall and Jones (2009) who note that the period from veraison to maturity is particularly important for the production of desirable wine grapes. A long period and optimum temperature enables the fruit to develop flavours that add value to a finished wine. Night temperatures in particular have been found to be an important determinant of wine composition (Mori et al., 2005; Cohen et al., 2008; Gaiotti et al., 2018). In some cooler regions worldwide, the shortening of the period between budbreak and maturity due to global warming has actually led to an improvement in grape quality (Van Leeuwen and Darriet, 2016; Koch and Oehl, 2018).

The seasonal effects on phenology in this study were considerable. Maturity dates were up to 28 days apart from year to year and the number of days between budbreak and maturity being 30 days longer in both TH vineyards at DCY and DBR in the cool season of 2016–17 compared to the warmer seasons in 2015–16 and 2017–18 (Table 3). This shortening of time between phenological stages in warmer seasons is consistent with other studies (Jones et al., 2005; Malheiro et al., 2013). This is also consistent with studies into the effect of increased soil temperature on post-harvest carbohydrate accumulation (Holzapfel and Smith, 2012; Hall et al., 2016) and enhanced vegetative and reproductive growth (Field et al., 2009; Rogiers et al., 2011, 2014; Clarke et al., 2015).

Over the four years of the study, all budbreak and harvest dates were already in the projected MTA 1.26 or 2.61 ranges of Hall et al. (2016) at all sites except the highest (790 m) vineyard at DCW. This is in contrast with the findings of Alikadic et al. (2019) that with climate change, advances in phenology were more pronounced at higher elevation. There is some evidence that vines have different phenological behaviour at higher elevation sites (Caffarra and Eccel, 2010) where lower average temperatures could lead to phenotypical adaptation of growth rates. However, comparing the timing of phenological events at a particular vineyard with regionally calculated dates requires some caution. It has been useful to make a brief comparison to projected future phenology for the regions, however, no firm conclusions about long-term trends in phenological stages can be drawn as four years of data does not provide enough statistical power to allow definite determinations.

## Conclusion

The aim of this study was to compare climatic indices calculated from in-vineyard collected climate data (TT) and an interpolated climate dataset (specifically, SILO) for two spatially homogenous vineyards and two topographically diverse vineyards in three wine regions in Victoria over four growing seasons. The data retrieved from SILO for maximum temperatures generally correlated well with the data collected from the data loggers at all sites. There was also good correlation for minimum temperatures in the spatially homogenous vineyards but not in the spatially diverse vineyards where night-time thermal layering is likely to occur. Night temperatures are a significant determinant of grape composition. Hence, from a practical point of view, the use of the SILO data for the calculation of climatic indices in spatially homogenous vineyards in order to plan vineyard management for future climate scenarios or to investigate climate analogues or to predict phenological phases, can be considered to have similar accuracies to within-vineyard collected climate data. However, caution would need to be exercised by spatially diverse vineyards where cool air pooling occurs at night.

Even at topographically complex sites, knowledge of local conditions would allow interpretation of SILO derived indices in order to gain climate information unique to the site terroir, which would be more useful than published regionally derived indices. Due to the readily accessible, downloadable nature of the SILO data, this will allow any vineyard, anywhere in Australia to calculate their own 30-year average climatic indices and track these annually, providing them with an excellent tool for long-term vineyard management decisions, albeit with some interpretation required at hilly sites. Similar interpolated climate databases exist worldwide. Vineyards, or other agricultural enterprises, in other parts of the world could verify results in their location with the local interpolated climate database.

## Data Availability Statement

The raw data supporting the conclusions of this article will be made available by the authors, without undue reservation.

## Author Contributions

PP and AH conceived and designed the analysis with support from BH and SR. PP collected the data, performed the analysis with support from AH, and wrote the manuscript with input from BH, SR, and AH. AH contributed analysis tools. All authors contributed to the article and approved the submitted version.

## Conflict of Interest

The authors declare that the research was conducted in the absence of any commercial or financial relationships that could be construed as a potential conflict of interest.
